# RAG-2 deficiency results in fewer phosphorylated histone H2AX foci, but increased retinal ganglion cell death and altered axonal growth

**DOI:** 10.1038/s41598-019-54873-w

**Published:** 2019-12-06

**Authors:** Noemí Álvarez-Lindo, Jimena Baleriola, Vivian de los Ríos, Teresa Suárez, Enrique J. de la Rosa

**Affiliations:** 1 3D Lab: Development, Differentiation & Degeneration, Centro de Investigaciones Biológicas, Consejo Superior de Investigaciones Científicas (CIB/CSIC), Madrid, Spain; 2 Proteomics and Genomics, Centro de Investigaciones Biológicas, Consejo Superior de Investigaciones Científicas (CIB/CSIC), Madrid, Spain; 3 Present Address: Laboratory of local translation in neurons and glia, Achucarro Basque Center for Neuroscience; Department of Cell Biology and Histology, University of the Basque Country, Leioa; and Ikerbasque Foundation, Bilbao Bizkaia, Spain

**Keywords:** Developmental neurogenesis, Neuronal development

## Abstract

DNA double-strand breaks (DSBs), selectively visualized as γ-H2AX^+^ foci, occur during the development of the central nervous system, including the retina, although their origin and biological significance are poorly understood. Mutant mice with DSB repair mechanism defects exhibit increased numbers of γ-H2AX^+^ foci, increased cell death during neural development, and alterations in axonogenesis in the embryonic retina. The aim of this study was to identify putative sources of DSBs. One of the identified DSBs sources is LINE-1 retrotransposition. While we did not detect changes in LINE-1 DNA content during the early period of cell death associated with retinal neurogenesis, retinal development was altered in mice lacking RAG-2, a component of the RAG-1,2-complex responsible for initiating somatic recombination in lymphocytes. Although γ-H2AX^+^ foci were less abundant in the *rag2*^*−/−*^ mouse retina, retinal ganglion cell death was increased and axonal growth and navigation were impaired in the RAG-2 deficient mice, a phenotype shared with mutant mice with defective DNA repair mechanisms. These findings demonstrate that RAG-2 is necessary for proper retinal development, and suggest that both DSB generation and repair are genuine processes intrinsic to neural development.

## Introduction

The assembly of a functional central nervous system (CNS) is a complex process that requires coordination between proliferation, differentiation, and programmed cell death during early stages of development^1–5^. In different areas of the CNS, including the hippocampus, frontal cortex, and retina, the transition from neuroepithelial cell to mature neuron coincides with massive cell death and an increase in the number of phosphorylated histone H2AX (γ-H2AX) foci, a selective marker of DNA double-strand breaks (DSBs)^1,6,7^. The generation of DSBs is immediately followed by the recruitment of γ-H2AX to induce active DNA repair, particularly that mediated by the nonhomologous end-joining (NHEJ) pathway. DNA repair is critical for proper neural development. Indeed, DNA repair defects usually lead to severe neural, as well as immune, alterations, in some cases resulting in embryonic lethality or microcephaly, while other tissues remain unaffected^3,8,9^.

A collection of genomic variations associated with somatic mosaicism in neurons, including deletions, insertions, copy number variations, and retrotransposition, have been described in the human and mouse CNS^10,11^. Most if not all of these DNA alterations depend, at some point, on DSB generation and repair pathways^12^. DSB repair is thus increasingly recognized as a fundamental element of CNS cellular dynamics during development and in adulthood. While the deleterious impact of unresolved DSBs in the CNS is well described^9,13–16^, how these DSBs are generated and their physiological role during neuronal differentiation remains poorly understood.

The mouse retina is a part of CNS that serves as a convenient model for the analysis of cellular processes involved in early neural development^16^. Particularly, the different phases of cell death that occur during retinal development are well characterized^1,17^. We previously described in the embryonic mouse retina an early phase of neural cell death during which γ-H2AX^+^ foci number increases, a peculiarity absent in later phases of retinal cell death^18^. One consequence of deficient DSB repair is programmed cell death; defects in NHEJ-mediated DSB repair result in increases in cell death that selectively affect recently born neurons, as described in the retinas of DNA-polymerase-µ- and Ku86-deficient mice, and SCID (DNA-PK) mutant mice^18–20^, as well as in the cerebral cortex of NHEJ-1-deficient mice^7^.

While proliferative and metabolic stressors have been proposed to give rise to DNA damage and DSBs^21^, other potential sources in the nervous system include active mechanisms related to the generation of neuronal somatic mosaicism^4,12^. To date, LINE-1 retrotransposition is the only DSB-generating mechanism identified in mouse neural tissues during development^22,23^. LINE-1 is an autonomous DNA retrotransposon that can replicate and intercalate itself into various locations within the genome, thereby generating DSBs and disrupting gene expression (reviewed in^23^). In human tissues, relative copy numbers of LINE-1 retrotransposon are higher in the brain than in non-neural tissue, although the role of LINE-1 retrotransposition in neural development remains controversial^24,25^. In addition to LINE-1, recent studies have characterized several endonucleases responsible for the generation of sequence-directed DNA breaks in adult neurons^26^ and other cell types^27^. In the immune system, the endonuclease RAG-1,2-complex is responsible for generating directed DSBs at sequence-specific targets and regulates DNA repair, primarily via the NHEJ pathway^28,29^. The RAG-1,2 functional complex consists of a catalytic subunit, RAG-1, and a regulatory subunit, RAG-2. RAG-1 is widely distributed throughout the nervous system^30^, and mutant mice defective for this subunit exhibit defects in memory formation^31^ and alterations in the olfactory system^32^. By contrast, RAG-2 is expressed at low levels in the zebrafish nervous system, except for the olfactory bulb^33^, and *rag2* mRNA expression has been reported in the mammalian brain and retina^34,35^.

Here, we demonstrate that RAG-2 protein is present in the embryonic mouse retina and is involved in early retinal development. The absence of RAG-2 in the embryonic retina increases cell death at E13.5 and leads to abnormal axonal growth, supporting that RAG-2 is required for proper retinal development in mice.

## Results

### Possible sources of DSBs in the developing mouse retina

**Figure 1:**
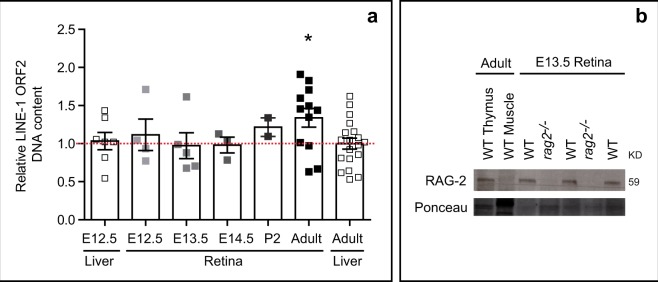
Possible sources of DSBs in the developing retina. (**a**) Relative levels of LINE-1 DNA detected by genomic qPCR in WT mouse liver and retinal extracts collected at different developmental stages and in adulthood. Dotted red line indicates the mean LINE-1 DNA content in the adult liver. Each datapoint represents a pool of littermates in the case of embryonic tissue samples, and a single animal in the case of adult tissue samples (only 2 animals in P2). Histograms depict the mean ± SEM. *P < 0.05 vs. LINE-1 adult liver content. **(b**) Western blot analysis of RAG-2 protein levels in retinal extracts from E13.5 WT and *rag2*^*−/−*^ mice. WT adult thymus was used as positive control; WT adult muscle was used as negative control. E, embryonic day; P, postnatal day.

The origins of DSBs in the developing retina remain unclear^18–20,36^. Characterization of potential sources of DSBs could provide important clues as to their physiological relevance. To investigate the role in retinal development of LINE-1, a putative source of DSBs, we measured the relative levels of its *orf2* sequence by genomic quantitative PCR (Fig. 1a).

We observed no significant changes in relative amounts of LINE-1 between E12.5 and E14.5, the period during which γ-H2AX^+^ foci incidence peaks and retinal ganglion cells (RGCs) are generated and selectively targeted by programmed cell death^18^. Genomic levels of LINE-1 in the developing embryonic retina were comparable to those found in both the embryonic and adult liver (Fig. 1a). Significantly higher levels of LINE-1 were detected in the adult retina, an interesting observation that is beyond the scope of this study.

Based on these findings, which suggest that LINE-1 is not involved in the generation of DSBs during this stage of retinal development, we investigated the RAG-1,2-complex, which exerts an essential endonuclease activity in the immune system^37^, as a possible source of DSBs during early retinal development. The detection of RAG-2 protein in the E13.5 WT retina (Fig. 1b), together with previous reports of RAG-1 expression in the retina^30–32,38^, establishes the expression of the two subunits known to be required for stable DNA binding and cleavage activity in the immune system and suggests a physiological function involving RAG-1,2-complex endonuclease activity in DSB generation in the developing retina.

### RAG-2 is active in the E13.5 mouse retina

**Figure 2:**
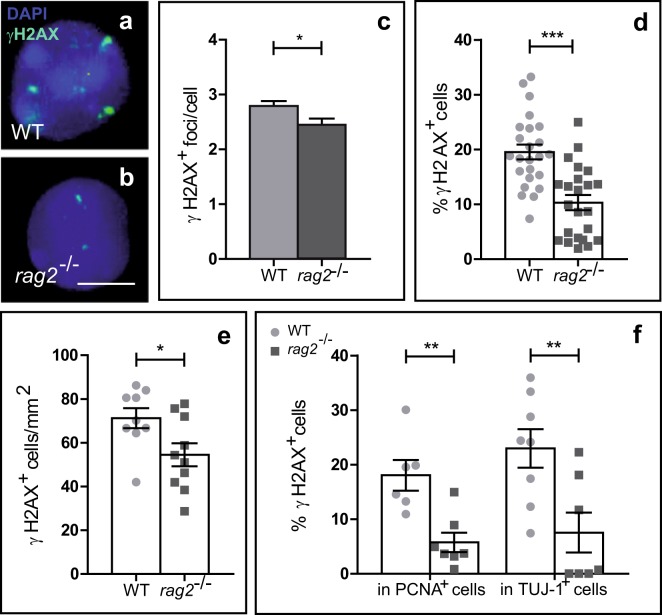
DSB number is reduced in the retinas of E13.5 *rag2*^*−/−*^ mice. (**a,b**) Presence of DSBs in WT and *rag2*^*−/−*^ mice as determined by γH2AX immunostaining (green). Nuclei were counterstained with DAPI (cyan). Quantification of nuclear γH2AX positive foci per cell (**c**) and percentage of γH2AX^+^ cells **(d)** in dissociated retinal cells from WT and *rag2*^*−/−*^ mice (n > 100). **(e)** Density of γH2AX^+^ cells in whole-mount retinas. (**f**) Immunostaining for γH2AX, PCNA, and TUJ-1 in dissociated retinal cells from WT and *rag2*^*−/−*^ animals. The percentage of γH2AX^+^ proliferative cells (PCNA^+^) and γH2AX^+^ neurons (TUJ-1^+^) are shown. Histograms depict the mean ± SEM values. *P ≤ 0.05, **P ≤ 0.01, ***P ≤ 0.001 vs. corresponding controls. Individual values are depicted as circles (WT) and squares (*rag2*^*−/−*^). Scale bar, 5 µm in a and b.

To confirm that RAG-2 has a function in retinal development, we compared *rag2*^*−/−*^ and WT retinas (Fig. 2). Immunostaining for γ-H2AX in retinal cells dissociated at E13.5 revealed the presence of the characteristic γ-H2AX^+^ foci associated with the presence of DSBs^39^ (Fig. 2a,b). γH2AX immunostaining was reduced in *rag2*^*−/−*^ versus WT retinas (Fig. 2), an observation that correlates with its expected role as endonuclease. In dissociated retinal cells from RAG-2-deficient mice the number of γ-H2AX^+^ foci per cell and the number of cells containing γ-H2AX^+^ foci were reduced as compared with WT controls (Fig. 2c,d). In line with this observation, the density of γ-H2AX^+^ cells in whole-mount retinas was lower in *rag2*^*−/−*^ than WT retinas (Fig. 2e). This decrease was evident in the two cell populations found in the E13.5 retina, i.e., PCNA^+^ proliferative neuroepithelial cells and recently differentiated TUJ-1^+^ neurons (Fig. 2f). These results support the involvement of RAG-2 in retinal development. Although our experimental approach allowed only indirect assessment, our findings are compatible with the interpretation that RAG-2 is at least partially responsible for the generation of naturally occurring DSBs in the developing retina^18–20,36^.

### RGC death is increased in the E13.5 mouse retina in the absence of RAG-2

**Figure 3:**
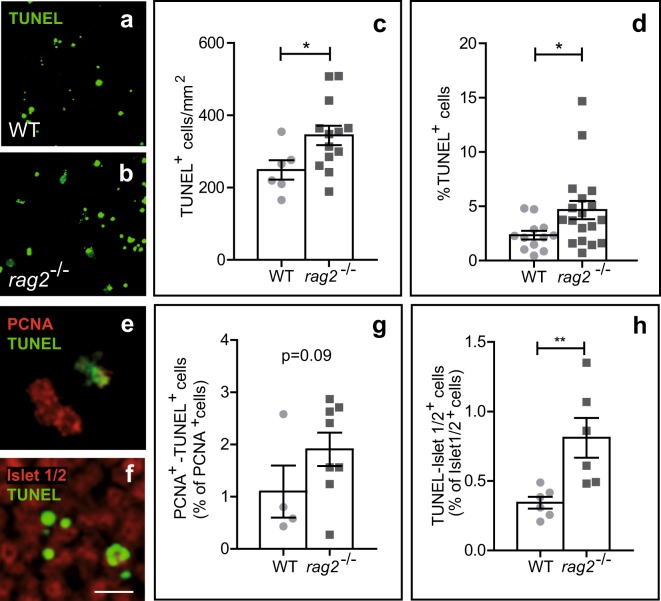
Neuronal cell death is increased in E13.5 *rag2*^*−/−*^ retinas. (**a,b,e,f**) Apoptotic cells as visualized by TUNEL (green) in WT and *rag2*^*−/−*^ mouse retinas. Density of TUNEL^+^ nuclei in whole-mount retinas (**c**) and percentage of TUNEL^+^ cells in dissociated retinal cells (**d**) are shown. (**e,g**) Dissociated retinal cells were immunostained for PCNA (red) and processed for TUNEL (green), and labelled cells were scored. (**f,h**) Apoptotic neurons were scored in whole-mount retinas immunostained for Islet 1/2 (red) and processed for TUNEL. Histograms show the mean ± SEM values. *P < 0.05, **P < 0.01 vs. corresponding controls. Individual values are depicted as circles (WT) and squares (*rag2*^*−/−*^). Scale bar, 50 µm in a and b, 12 µm in e and f.

We next examined the effect of RAG-2 deficiency on retinal neurogenesis, specifically during the early neural cell death phase, which affects recently born RGCs^1,17^. Compared with WT retinas, the number of apoptotic TUNEL^+^ cells was significantly higher in both whole-mount retinas (Fig. 3a–c) and dissociated cells (Fig. 3d) from *rag2*^*−/−*^ mice, even though RAG-2 deficiency was associated with fewer γ-H2AX^+^ foci (Fig. 2). Double TUNEL and PCNA immunostaining of dissociated retinal cells revealed no significant differences in numbers of dying neuroepithelial cells between WT and *rag2*^*−/−*^ mice (Fig. 3e,g). Conversely, TUNEL combined with specific Islet-1/2 immunostaining of RGCs at this embryonic stage revealed increased cell death in this population of young neurons in *rag2*^*−/−*^ versus WT mice (Fig. 3f,h).

Contrary to expectations, the neuronal cell death phenotype observed in RAG-2-deficient mice, in which the number of γ-H2AX^+^ foci is reduced versus WT (Fig. 2), was in line with that described in DNA-PK (*SCID*) and DNA polymerase μ (*polμ*^*−/−*^) DSB repair mutants, which accumulate γ-H2AX^+^ foci^18,19^. Based on this observation, we next sought to characterize other phenotypic consequences of RAG-2 deficiency.

### RGC axonal growth is altered in RAG-2-deficient mice

**Figure 4:**
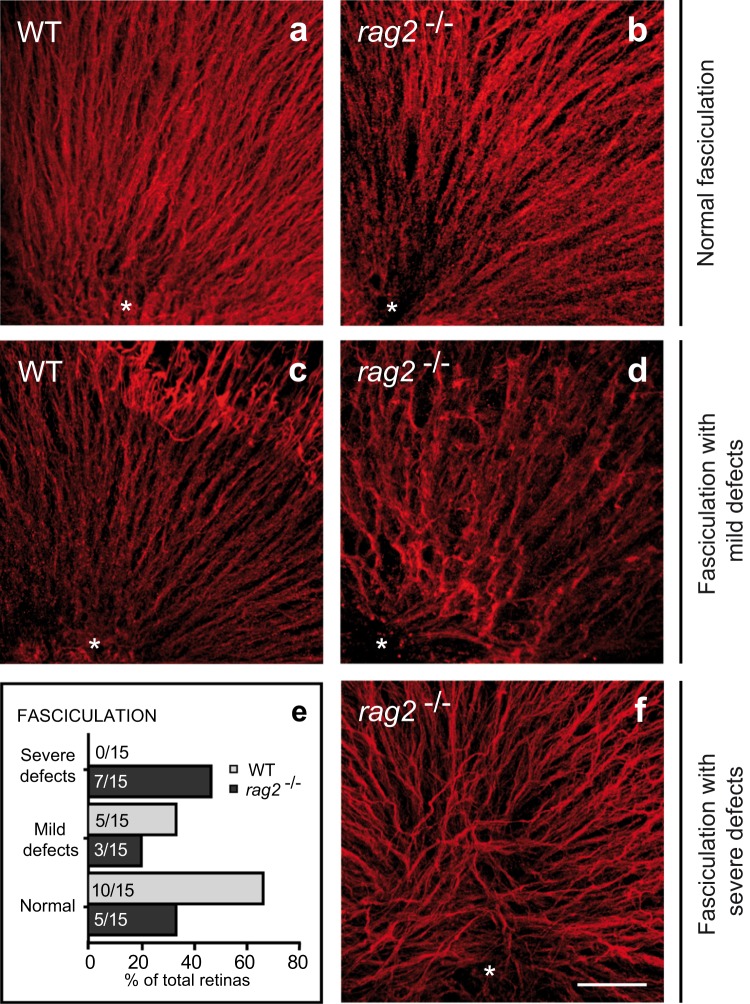
*In vivo* axonal navigation is altered in E13.5 *rag2*^*−/−*^ mice. RGC axonal trajectories were visualized by TUJ-1 immunostaining in whole-mount retinas from E13.5 WT (**a**,**c**) and *rag2*^*−/−*^ mice (**b,d**,**f**). The optic nerve head is indicated with an asterisk. According to the axonal trajectory observed, fasciculation was classified as normal **(a-b)**, mildly defective (**c,d**), or severely defective (**f**). The histogram (**e**) depicts the proportion (%) and absolute number (indicated within each bar) of retinas for which each phenotype was observed. Scale bar, 50 µm in a-d and f.

RGC axonal growth is impaired in mice that lack repair DNA polymerase μ (Pol μ)^19^. We therefore investigated whether RAG-2 deficiency gives rise to a similar phenotype. Intraretinal axonal trajectories were visualized in E13.5 WT and *rag2*^*−/−*^ mice by TUJ-1 immunostaining of retinal whole-mounts (Fig. 4a–d,f). WT retinas typically exhibited regular fasciculation, with axons converging at the optic nerve head (Fig. 4a,c). By contrast, *rag2*^*−/−*^ mouse retinas showed alterations in axonal growth, including markedly disorganized fasciculation, reduced axonal density, and tangential axonal trajectories (Fig. 4b,d,f). According to the images of TUJ-1-immunostained retinas, fasciculation was classified as normal when ≥90% of the image area showed regular fasciculation; as mildly defective, when axonal density was reduced below 90%; or as severely defective, when besides a reduction in axonal density, tangential axonal trajectories were observed (quantified in e). Moreover, we also observe that midline crossing of optic fibres in E13.5 *rag2*^*−/−*^ mice was delayed relative to WT counterparts (Supp. Fig. 1), but the distribution of cell adhesion molecules involved in axonal guidance, namely L1-CAM and Bravo, that were altered in the *polμ*^*−/−*^ mutant mice^19^, were not affected (data not shown).

To further characterize the axonal disturbances observed in the *rag2*^*−/−*^ mouse retina, we performed 2D electrophoresis and protein identification in E15.5 retinal extracts from WT and *rag2*^*−/−*^ mice. Significant alterations (p < 0.05; Supp. Fig. 2 and Table 1) in the protein levels of tubulin β3 and tubulin α1C, constituents of the axonal cytoskeleyon, fascin and platelet-activating factor acetylhydrolase 1, also implicated in axonal function, were observed in the mutant retinas supporting the view that RAG-2 deficiency affects axonal structure and function (Table 1).

**Table 1:** Proteins for which alterations in expression were observed in *rag2*^*−/−*^ *versus* WT mouse retinas, and corresponding roles in axonal function.

Protein name	Identifiers and Chemical properties		Matching		Relation to axonal function
Tubulin, a1C	*# Spot*^*a*^	SSP4602	*Peptides matched*^*f*^	24	Tubulins are microtubule elements, being the isoform b3 almost specific of neurons. Tubulins a1 and b3 are proteins implied in citoskeletal structures that coordinate motility and cell adhesion^60^. Defects in these proteins result in altered axon guidance and neuronal migration^61–63^.
	*Acc. Code*^*b*^	gi|6678469			
	*Mr (Da)*^*c*^	50562	*Mascot total score*^*e*^	404	
	*pI*^*d*^	4.78	*% Seq. Coverage*^*g*^	49	
Tubulin, b3	*# Spot*^*a*^	SSP4602	*Peptides matched*^*f*^	13	
	*Acc. Code*^*b*^	gi|12963615			
	*Mr (Da)*^*c*^	50418	*Mascot total score*^*e*^	94	
	*pI*^*d*^	4.82	*% Seq. Coverage*^*g*^	23	
Fscn1 protein	*# Spot*^*a*^	SSP6601	*Peptides matched*^*f*^	19	Fascin1 is a bundling protein present in actin filaments. It is implied in cytoskeletal structures that coordinate motility and cell adhesion^60^.
	*Acc. Code*^*b*^	gi|144719132			
	*Mr (Da)*^*c*^	52157	*Mascot total score*^*e*^	106	
	*pI*^*d*^	6.57	*% Seq. Coverage*^*g*^	32	
Pafah1b3	*# Spot*^*a*^	SSP304	*Peptides matched*^*f*^	6	Platelet-activating factor acetylhidrolase 1 (Pafah1), formerly known as Lissencephaly-1, also participates in axon guidance and neuronal migration during embryonic development interacting with dinein at microtubules^64,65^.
	*Acc. Code*^*b*^	gi|6679201			
	*Mr (Da)*^*c*^	259551	*Mascot total score*^*e*^	93	
	*pI*^*d*^	6.42	*% Seq. Coverage*^*g*^	24	

The proteins depicted correspond to the most statistically significant protein candidates encoded in the *Mus musculus* proteome. The data were provided by Mascot search engine. ^a^Spot numbering as shown in the 2D-gels depicted in Supp. Fig. 2. ^b^Protein accession code from *Mus musculus* database. ^c^Theoretical molecular weight (Da). ^d^Theoretical isoelectric point (pI). ^e^Mascot Total score is −10*Log(P), where P is the probability that the observed match is a random event. ^f^Number of matched peptides. ^g^Sequence coverage is the ratio of amino acids (no. identified in peptides/no. in theoretical peptides from sequence data) expressed as a percentage.

### Neurite outgrowth is dysregulated in dissociated retinal cell cultures from RAG-2-, Pol μ-deficient, and DNA-PK-mutant mice

**Figure 5:**
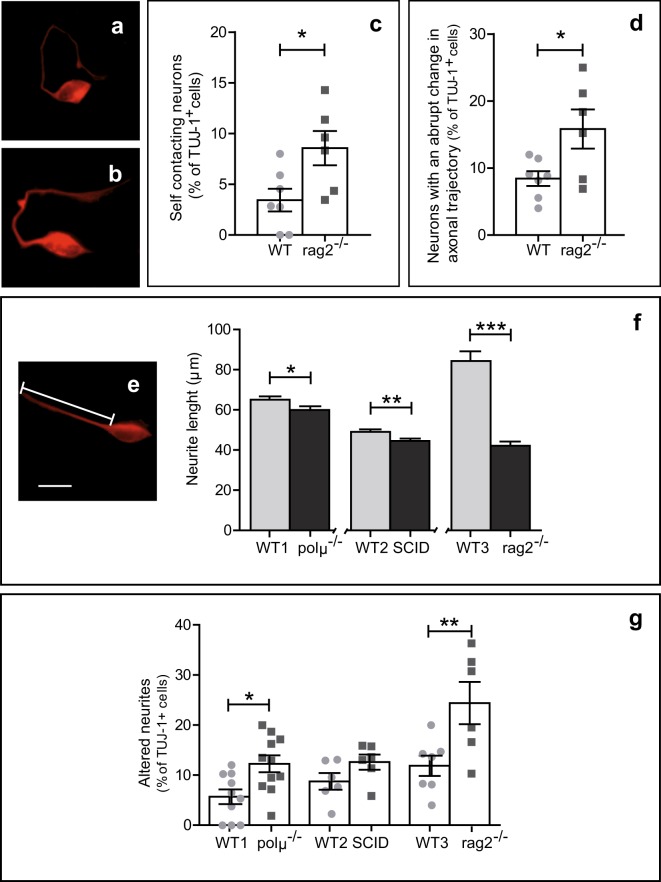
Altered neurite growth in E13.5 *rag2*^*−/−*^*, SCID*, and *polμ*^*−/−*^ cultured retinal cells. E13.5 dissociated retinal cells from the indicated mouse genotypes were cultured on surfaces pretreated with polyornithine and laminin. Neurons and their neurites were visualized by TUJ-1 immunostaining and classified based on trajectory as straight (**e**), self-contacting (**a**), or with abrupt changes in their directionality (**b**), and plotted by typology (**c,d**). Neurite length (**e**) was measured in retinal cells from *rag2*^*−/−*^, *pol*μ^*−/−*^, and *SCID* mutant mice after culture for 18 hours and compared with that of WT counterparts (WT1-C57BL/6; WT2-Balb/C; WT3-C57BL/10) (n > 100 cells) (**f**). The total percentage of neurites exhibiting alterations in directionality was determined in retinal cultures from the 3 mutant mice and compared with that observed in corresponding WT controls (n > 100 cells) (**g**). Percentages in c, d and g refer to the number of TUJ-1^+^ cells emitting neurites. Histograms show the mean ± SEM. *P < 0.05, **P < 0.01, **P < 0.001 vs. corresponding controls. Individual values are depicted as circles (WT) and squares (mutant mice). Scale bar, 10 µm in a, b and e.

Axonal growth and navigation is a complex process mediated by interactions between environmental cues and cell autonomous determinants^40^. Based on our previous observation of abnormal neurite outgrowth in dissociated *polμ*^*−/−*^ retinal cells^19^, we examined neurite phenotype in *rag2*^*−/−*^ retinal cells (Fig. 5). To minimize the influence of neighbouring cells on axonal growth, dissociated retinal cells from E13.5 WT and *rag2*^*−/−*^ mice were cultured at low density in homogeneous, defined media. After culture for 18 hours the proportions of neurite-bearing cells among isolated TUJ-1^+^ cells were comparable in WT and *rag2*^*−/−*^ mice (62.9 ± 6.1% and 58.1 ± 5.9% of total TUJ-1^+^ cells, respectively; n = 6 litters). However, neurite morphology differed between the groups; while over half of WT TUJ-1^+^ cells emitted straight unbranched neurites (53.7 ± 6.2% of labelled cells), the proportion of these cells was lower in *rag2*^*−/−*^ TUJ-1^+^ cells (41.8 ± 10.3%). RAG-2-deficient neurons more frequently had morphologically aberrant phenotypes, including self-contacting neurites (Fig. 5a,c), as well as abrupt changes in neurite trajectory (Fig. 5b,d). Interestingly, the presence of aberrant and shorter neurites was common to cultured *rag2*^*−/−*^, *polμ*^*−/−*^, and *SCID* retinal neurons (Fig. 5e–g), although in the case of *SCID* retinal cells a significant difference relative to WT controls was observed only in neurite length (Fig. 5f). These data altogether define a cell-autonomous phenotype related to axonal growth and navigation that is common to *rag2*^*−/−*^, *polμ*^*−/−*^, and *SCID* mouse retinas, despite the opposite role of these proteins on DSB generation (RAG-2) and repair (Pol μ and DNA-PK).

Together, our results support the involvement of the endonuclease RAG-2 in early retinal development, and suggest direct or indirect roles in DSB generation, the early phase of neural cell death, which affects RGC generation, and cell autonomous control of RGC axonal growth.

## Discussion

Our previous studies have demonstrated that DSBs, as visualized by γ-H2AX immunostaining, are generated during the early stages of retinal development, and have characterized the impact of deficient DNA repair mechanisms on RGC survival and axonal growth in *SCID* and *pol*μ^*−/−*^ mutant mice^18,19^. In this study, we sought to characterize sources of DSBs and determine their physiological relevance.

Evidence suggests that LINE-1, retrotransposition of which gives rise to DSBs, may be involved in the generation of somatic mosaicism in the nervous system^22,23,41,42^. We found that relative amounts of LINE-1 DNA were modestly but significantly increased in the adult retina, but not during retinal neurogenesis, ruling out LINE-1 as a relevant source of the observed γ-H2AX^+^ foci^18^ in retina at E13.5. Widespread expression of the RAG-1 subunit in the nervous system, including the retina, had been previously described^30,38^, while nervous system expression of RAG-2 was poorly characterized^34,35^. Our analyses revealed the presence of RAG-2 protein in retina at E13.5, an observation suggestive of a physiological role in DSB generation. Our finding is compatible with the formation of RAG-1,2-complexes in the developing retina, a situation that has not been described in non-immune cell types^30,33,43,44^. In line with the demonstrated role of the RAG-1,2-complex in the immune system, we observed fewer γ-H2AX^+^ foci in RAG-2-deficient versus WT retinas. Our observations in the *rag2*^*−/−*^ mouse retina provide for the first time clear evidence of a role of RAG-2 in retinal development.

Our previous studies confirmed that one direct consequence of deficient DSB repair is increased cell death, which in the developing retina particularly affects RGCs, the first neuronal cell type generated during retinal neurogenesis^18,19^. However, this causal relationship is called into question by our findings in RAG-2-deficient mice. In the E13.5 *rag2*^*−/−*^ mouse retina we observed a decrease in γ-H2AX^+^ foci number that were compatible with reduced endonuclease activity, but, unexpectedly, increased numbers of dying RGCs. Furthermore, altered RGC axonal growth and navigation, the characteristic phenotype of DSB repair mutants, were also observed in RAG-2-deficient retinas, specifically alterations in axonal growth and intra- and extra-retinal axonal navigation, as well as cell-autonomous defects in neurite outgrowth. The selective RGC death in the *rag2*^*−/−*^ mice, in contrast to the non-significantly affected population of proliferative neuroepithelial cells, might point to the existence of a still unknown selective mechanism active on RGCs, essential for their correct development and the generation of neuronal diversity, as has been previously proposed^12^.

There are several possible direct and indirect mechanisms that could link RAG-2 deficiency with defects in RGC development. Increased RGC death could affect axonal pathfinding, since some axonal guidance cues are exposed on the axonal surface of pioneer or surrounding neurons^40,45^. This hypothesis, which can also be applied to *SCID* and *polμ*^*−/−*^ mutant mice, does not explain the cell-autonomous neurite phenotype observed in dissociated retinal-cell cultures from *rag2*^*−/−*^ mice (Fig. 5). Although beyond the scope of this study, another potential explanation is that neural development of these mutants is impaired as a consequence of maternal immunodeficiency^46,47^ caused by the absence of RAG-2, DNA-PK, or Pol µ^48–50^.

The coupling of DNA endonuclease activity and DNA repair mechanisms to generate cellular diversity may not be exclusive to the immune system, and may be a feature of other biological processes requiring programmed DNA cleavage. It is tempting to speculate that RAG-2-mediated DSBs promote the acquisition of somatic alterations in recently born RGCs. This phenomenon, mediated by the RAG-1,2-complex and NHEJ repair mechanisms, is well established during lymphocyte generation and diversification^37,51^. The RAG-1,2-complex binds DNA in specific sequences, at canonical sites within immunoglobulin genes or lymphocyte receptor gene rearrangement sites (RSSs). However, cryptic, non-canonical RSSs are spread throughout the genome and, at least in lymphocytes, usually correspond to potential RAG-1,2 binding and cutting sites^52^, although off-target endonuclease activity in immune cells is generally scarce^52,53^. It should be noted that, according to the RSS-site database^54^, the 4 axonal proteins for which we observed altered expression in *rag2*^*−/−*^ mice (Table 1) contain putative RSSs that can be recognized by the RAG-1,2-complex endonuclease, supporting a possible direct role of RAG-2 in axonogenesis and, maybe, axonal guidance. These observations, taken together with the alterations in retinal development observed in RAG-2-deficient mice and the somatic mosaicism described in the brain^10,12^, make it tempting to propose a role for RAG-2-mediated DNA breaks in the generation of neuronal mosaicism in the retina, and a role of mosaicism in proper neural connectivity. In line with this view, a recent study has proposed that DSBs may play important roles in brain physiology and disease, and research attention is beginning to focus on the mechanisms responsible for DSB generation and repair^55^.

In summary, our findings in the *rag2*^−/−^ mouse underscore the importance of this component of the DNA cleavage mechanism in RGC viability and axonogenesis. These data, together with previous findings in DNA-repair-deficient mice, describe a possible scenario in which the RAG-1,2-complex mediates DSB generation and induces NHEJ repair during retinal development. Further studies employing single-neuron whole-genome sequencing will be required to establish a direct link between those processes and neuronal diversification, as already demonstrated in the immune system.

## Materials and Methods

### Mice

All experimental procedures in this paper were performed in accordance with European Union regulations for the use and treatment of animals in research (RD53/2013, BOE, Spain), and were approved by the CSIC bioethics committee for animal experimentation and the Dirección General de Medio Ambiente, Comunidad de Madrid. Male and female mouse embryos and adult tissues were used in all experiments. C57BL/10SnJ^rag2*−/−*^ mice (*rag2*^*−/−*^; Taconic, Hudson, NY, USA) were provided by Dr. J.A. García-Sanz (CIB) and Balb/C-JHA^hsd-prkdc SCID^ (*SCID*) mice were obtained from Harlan (Gannat, France). DNA-polymerase-mu-deficient mice (*polμ*^*−/−*^) were kindly provided by Dr. Luis Blanco and backcrossed to a C57BL/6 J background as described in^19^. Three different wild type strains (WT) were used, according to the three different mutants respective genetic background: C57BL/10JCrl mice were obtained from Charles River (Ecully, France), while Balb/C JHA and C57BL6/J were both obtained from Harlan. WT and mutant mice were bred in local facilities and euthanized at the indicated ages.

### LINE-1 levels in tissue

LINE-1 levels were determined by quantitative PCR of genomic DNA from liver and retina. SYBR-green qPCR assays were performed using 0.1 ng of genomic DNA and primers against 5 S DNA (normalizer gene) and LINE-1 *orf2* (target gene). PCR was performed using the following conditions: 95 °C for 10 min followed by 40 cycles of 95 °C for 30 s, 60 °C for 30 s, and 72 °C for 1 min. The following primers were used: 5 S.2, 3′ GCCTACAGCACCCGG; 5 S.3, 5′ GTCTACGCCATACCACC; L1.1, 5′ TAAAGAACTCAAGAAGGTGG; L1.2, 3′ AGGGTTGTTTTGATTTGC. Data were analyzed using the ΔCt method relative to the normalizer gene^56^. LINE-1 levels in the different tissues were compared with those found in the adult liver.

### Western blot

Individual adult tissues and embryonic retinas were processed as previously described^19^. Briefly, tissues or retinas were lysed, and 50 μg of total protein was resolved by PAGE and transferred to PVDF membranes. Membranes were blocked and incubated with antibodies against RAG-2 (1/1000, Abcam #ab133609; Cambridge, UK), and subsequently with a polyclonal goat anti-rabbit HRP secondary antibody (Dako Cytomation, Glostrup, Germany).

### Immunostaining of dissociated cells

Freshly dissected retinas from E13.5 embryos were dissociated and the dissociated cells were fixed onto glass slides by cytospin as previously described^18^. Cytospin slides were incubated overnight at 4 °C with primary antibodies against γH2AX (1/1000, Ser 139, Abcam #ab22551); human β-III tubulin clone TUJ-1 (1/1000, PRB-435P; Covance, Paris, France); and PCNA [1/250, PCNA(C19) antibody, DB051; Delta Biolabs, Gilroy, CA, USA] and then for 1 h at room temperature (RT) with Alexa Fluor-conjugated secondary antibodies (1/250–1/500, depending on the primary antibody; Molecular Probes/Thermo Scientific, Rockford, IL, USA). Cell death was detected using the TUNEL technique (TdT-mediated dUTP nick end-labelling of fragmented DNA; Promega, Madison, WI, USA) according to manufacturer instructions, and combined with immunohistochemistry when necessary. Preparations were counterstained with DAPI and mounted with Fluoromount-G mounting medium (Southern Biotech, Birmingham, AL, USA). Scoring of 100–500 cells from non-adjacent fields was performed using a fluorescence microscope (Zeiss Axioplan, Oberkochen, Germany) coupled to a CCD camera (Leica, Wetzlar, Germany) or a confocal microscope (Leica TCS-SP5-A0BS) with a 40X objective.

### Immunostaining and TUNEL analysis of whole-mount retinas

Freshly dissected E13.5 retinas were flat-mounted onto nitrocellulose membranes. Tissue fixation, permeation and antigen blocking were performed as previously described^18^. Retinas were immunostained with primary antibodies against γH2AX (1/1000), β-III tubulin (1/1000) or Islet-1/2 (1/200; Developmental Studies Hybridoma Bank #39.4D5, Iowa city, IA, USA), and then incubated for 1 h at RT with Alexa Fluor-conjugated secondary antibodies. The retina preparations were mounted with Fluoromount-G mounting medium and analyzed by confocal microscopy with a 40X objective. Cell death was detected using the TUNEL technique, combined with immunohistochemistry when necessary. Samples were counterstained with DAPI, and the density of TUNEL-positive cells was determined under a fluorescence microscope using a 100X objective. In all other cases, cell density was determined using FIJI software by calculating the number of cells present in 4 confocal images (40X) per retina, and normalizing according to the size of the imaged area.

### Primary dissociated neuroretina cell culture

Neuroretinas were dissected from E13.5 embryos, pooled per litter, and dissociated as previously described^18,19^. Dissociated cells from 3 different litters were plated in DMEM/F12 medium (Gibco, Life Technologies, Rockford, IL, USA) with N2 supplement at a density of 85,000 cells/cm^2^ in 2-cm^2^ Permanox chamber slides (NUNC, Thermo Fisher Scientific, Waltham, MA, USA) pre-treated with 0.5 mg/mL polyornithine (Sigma-Aldrich, San Luis, MI, USA) and 1 μg/mL laminin (Sigma-Aldrich), as previously described^19^. After incubation for 18–24 h at 37 °C and 5% CO_2_, cells were fixed for 20 min with 4% (w/v) paraformaldehyde (Sigma-Aldrich) in 0.1 M phosphate buffer (PB) at pH 7.4 and RT, and were processed for immunostaining as described above for dissociated cells.

### Anterograde DiI staining

E13.5 embryos were euthanized, the heads dissected out, and the lens removed from the eye cups, prior to fixation as previously described^20^. The optic nerve head was covered with Neurotrace DiI lipophilic marker (Molecular Probes) and, to ensure diffusion of the dye along the entire optic nerve, the heads were maintained in PB at 37 °C for 2 weeks. Optic nerve length and decussation at the optic chiasma were evaluated as previously described^19^.

### Two-dimensional gel electrophoresis and protein identification

For two-dimensional gel electrophoresis analysis, 25 μg of total protein from E13.5 mouse retina was loaded on ReadyStrip IPG strips for isoelectric focusing (linear 7-cm strips, pH 3–10; Bio-Rad, Hercules, CA, USA), following the manufacturer´s instructions. The strips were then equilibrated, applied to 12% SDS gels, and stained with SYPRO Ruby (Bio-Rad) following the manufacturer´s instructions. An EXQuest Spot Cutter was used for image acquisition and spot picking. The images were analyzed with PDQuestTM 2D Analysis 7.4 Software (Bio-Rad) as previously described^57^.

### MALDI peptide mass fingerprinting, MS/MS analysis and database searching

Spots of interest were manually excised and processed automatically in a Proteineer DP (Bruker Daltonics, Bremen, Germany). The sample was digested and prepared as previously described^58^, with minor modifications^59^. For MALDI-TOF/TOF analysis, samples were automatically acquired in an ABi 4800 MALDI TOF/TOF mass spectrometer (Applied Biosystems, Framingham, MA, USA) in positive ion reflector mode (ion acceleration voltage, 25 kV for MS acquisition and 1 kV for MS/MS). PMF and MS/MS fragment ion spectra were smoothed and corrected to zero baseline using routines embedded in ABi 4000 Series Explorer Software v3.6. Each PMF spectrum was internally calibrated with the mass signals of trypsin autolysis ions to reach a typical mass measurement accuracy of <25 ppm. GPS Explorer v4.9 was used to submit the combined PMF and MS/MS data to MASCOT software v.2.1 (Matrix Science, London, UK), searching in the non-redundant NCBI protein database. The mascot total score is −10*Log(P), where P is the probability that the observed match is a random event. The percentage of sequence coverage was calculated as the ratio of amino acids (number identified in peptides / number of theoretical peptides from sequence data).

### Statistical analysis

Animals from at least 3 independent litters were used for each experiment, unless indicated. All experiments were conducted at least twice. Data size was estimated in accordance with previous literature to fulfil the 3 R principles for animal experimentation. Bars in graphs represent the mean and the standard error of the mean, and data points represent individual mice or pools from a given litter, as indicated. In all cases, data were checked for normality using D’Agostino-Pearson omnibus and Shapiro-Wilk normality tests sequentially, and considered to fit a normal distribution only if they passed both tests. For normal data, homoscedasticity was determined using Fisher’s test. Normal data were compared using an unpaired Student’s T-test, applying Welch’s correction in cases of non-homoscedasticity. Non-normal samples were compared using the Mann Whitney nonparametric U-test. All analyses were performed at a fixed 95% confidence interval and outliers were excluded from further analysis according to Grubbs’ outlier test. Data analyses were performed using GraphPad Prism version 5.01 for Windows (GraphPad Software, San Diego, CA, USA). Statistically significant differences are indicated as follows: *p < 0.05; **p < 0.01; ***p < 0.001.

## Supplementary information

Supplementary figures
