# Supplementary material for: RAG-2 deficiency results in fewer phosphorylated histone H2AX foci, but increased retinal ganglion cell death and altered axonal growth
Source: Sci Rep. 2019 Dec 6;9:18486. doi: 10.1038/s41598-019-54873-w (PMC6898044; doi:10.1038/s41598-019-54873-w)
Supplement: Supplementary file 1 — Supplementary figures [file 41598_2019_54873_MOESM1_ESM.pdf]

RAG-2 deficiency results in fewer phosphorylated histone H2AX foci, but increased retinal ganglion cell death and altered axonal growth.

Álvarez-Lindo, Noemí[a,†], Baleriola, Jimena[a,c,†], de los Ríos, Vivian[b], Suárez, Teresa[a,*] and de la Rosa, Enrique J.[a,*]

[a]3D Lab: Development, Differentiation & Degeneration, Centro de Investigaciones Biológicas, Consejo Superior de Investigaciones Científicas (CIB/CSIC), Madrid, Spain.

[b]Proteomics and Genomics, Centro de Investigaciones Biológicas, Consejo Superior de Investigaciones Científicas (CIB/CSIC), Madrid, Spain.

[c]Current address: Laboratory of local translation in neurons and glia, Achucarro Basque Center for Neuroscience; Department of Cell Biology and Histology, University of the Basque Country, Leioa; and Ikerbasque Foundation, Bilbao, Bizkaia, Spain.

[†,*]Equal contribution

Correspondence:

Enrique J. de la Rosa, CIB, CSIC, Madrid, Spain; ejdelarosa@cib.csic.es

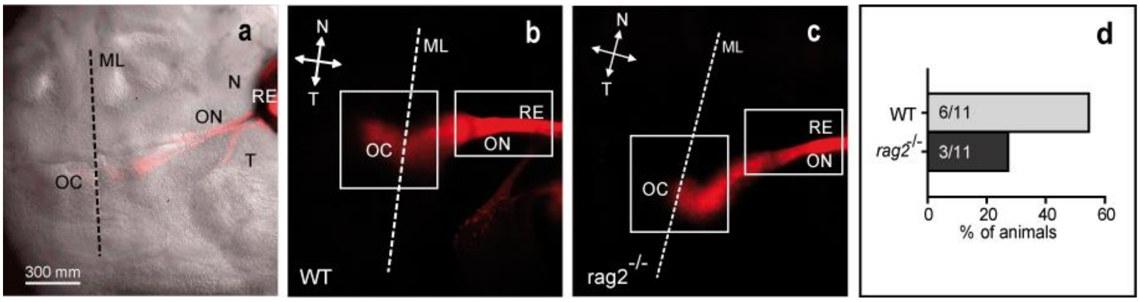

Supplementary Figure 1. Midline crossover in E13.5 *rag2$^{-/-}$* mouse.

(a-c) Anterograde DiI staining was used to visualize the optic nerve (ON) trajectory from the retina (RE) towards the optic chiasm (OC) at the diencephalon midline (ML) in E13.5 mice (N, nasal; T, temporal). **(b** and **c)** Midline crossover was evaluated in WT and *rag2$^{-/-}$* embryos. **(d)** The histogram shows the proportion (%) and absolute numbers (indicated within each bar) of animals whose optic fibres had crossed over the ML by E13.5.

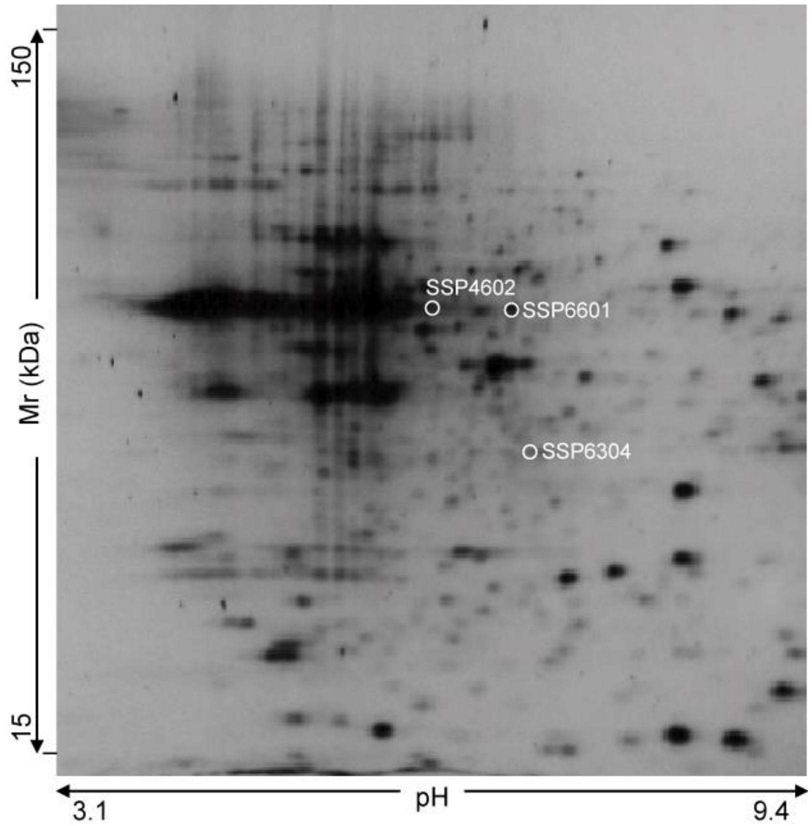

Spot No. [1]	Protein Name [2] [*Mus musculus*]	O.D. wt/ O.D. *rag2*$^{-/-}$ [3]
SSP4602	Tubulin, alpha 1C<br>Tubulin, beta 3	0.002
SSP6601	Fscn1 protein	20
SSP6304	Pafah1b3	200

Supplementary Figure 2. 2D-gel proteome analysis from E15.5 embryonic retina.

Retinal proteins in extracts from WT and *rag2*$^{-/-}$ mice were separated (250–10 kDa) using a 2D gel with a linear pH gradient (pH 3.0–10.0). Encircled spots indicate proteins for which changes in expression were observed between *rag2*$^{-/-}$ and WT mouse retinas. [1]Spot numbering as shown on the 2D gel. [2]Most statistically significant protein candidates encoded by the *Mus musculus* proteome, according to Mascot search engine. [3]Ratio of retinal WT:*rag2*$^{-/-}$ protein expression. O.D.; optical density.

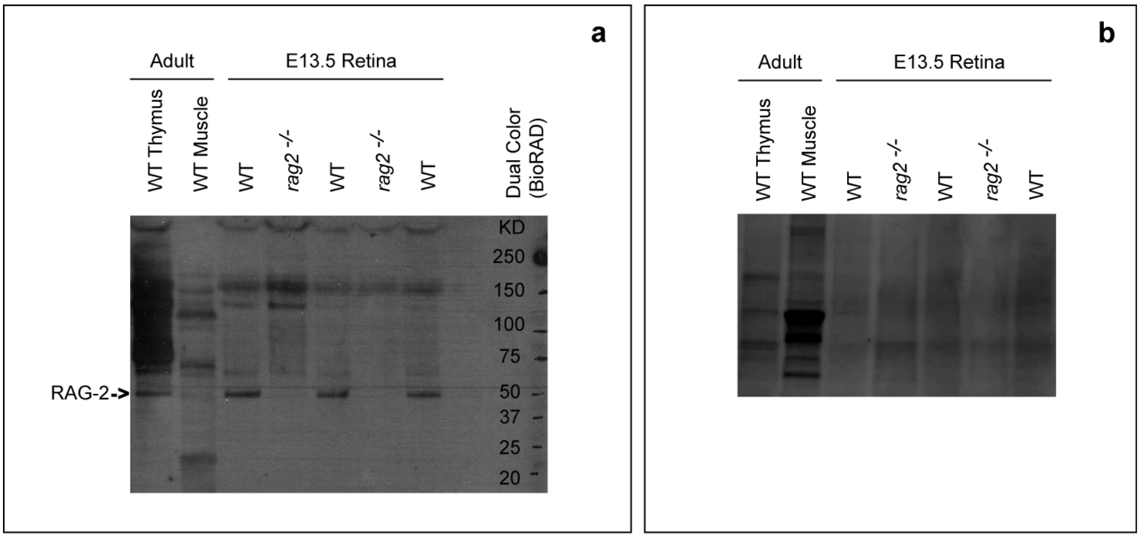

Supplementary Figure 3. Full western blot corresponding to images depicted in Figure 1b.

(a) Western blot was used to detect the RAG-2 subunit of RAG-1/2 complex in wild type E13.5 retinas. Adult muscle from wild type animals and retinas corresponding to E13.5 rag2-/- mice were used as negative controls. Thymus from wild type adult mice was used as positive control. **(b)** Ponceau staining from the same gel depicted in (a) (WT-Wild type)
